# Analysis of Popcorn (*Zea Mays* L. var. Everta) for Antioxidant Capacity and Total Phenolic Content

**DOI:** 10.3390/antiox8010022

**Published:** 2019-01-14

**Authors:** Michael G. Coco, Joe A. Vinson

**Affiliations:** Chemistry Department, Loyola Science Center, University of Scranton, 925 Ridge Row, Scranton, PA 18510, USA; mgcocojr@gmail.com

**Keywords:** Popcorn, polyphenols, antioxidant capacity, *Zea mays* L., pericarp, Folin–Ciocalteu, FRAP

## Abstract

Popcorn, one of the most popular snack foods in the world, is known for being a high fiber, healthy food. Our research has found that commercial popcorn also contains significant amounts of the class of antioxidants known as phenolic acids. The total antioxidant capacity of raw and popped popcorn extract has been quantified using the Folin–Ciocalteu and FRAP assays. The polyphenols were found exclusively in the pericarp of the kernel completely bound to the oligosaccharide fiber matrix. An in vitro digestion study was also performed to predict the phenolic acids’ bioavailability. On average, nine commercial popcorn samples contain 5.93 ± 0.92 mg/g of total polyphenols after alkaline hydrolysis and 2.66 ± 0.15 mg/g after in vitro digestion as measured by the Folin–Ciocalteu assay. Furthermore, the popping process was found not to significantly decrease the antioxidant capacity. These results indicate that a considerable amount of the bound polyphenols are bioaccessible. Due to the high levels of bioaccessible polyphenols, popcorn may be a significant source of dietary polyphenol antioxidants.

## 1. Introduction

Snack foods are generally thought negatively by nutritionists in that they usually have a high fat and sugar content. However, while this may be true of most snack foods, some have the potential to offer more to the consumer than just a simple hold over between meals. In fact, some studies have shown snacking to be positively associated with overall diet quality in adults and with consumption of whole grains [1]. One popular snack, popcorn, is known for being a healthy, whole grain alternative to other snacks such as potato chips.

According to the 2015–2020 Dietary Guidelines for Americans, at least half (about 3 oz) of one’s daily total grain intake should be whole grains [2]. However, while most Americans are reaching or exceeding the recommended 9 to 11 servings for grains eaten in one day, less than 8% of Americans eat the recommended minimum of 3 servings of whole grains in one day [2,3]. In Europe, daily consumption of whole grains varies greatly between countries with as little as 4 g/day in Italy and as high as 58 g/day in Denmark [4,5]. According to a consumption survey, snacks account for one-third of total whole grain consumption [6]. Eating whole grains has been linked to several health benefits including reducing the risk of chronic diseases including type 2 diabetes, coronary heart disease and hypertension [7,8,9].

The aim of this research was to determine the antioxidant content in several commercial popcorns. Plants are known to contain high amounts of bioactive compounds known as polyphenols, which studies have shown to have antioxidant activity [10]. Corn in general is known to be high in phenolic acids, with ferulic acid being predominate [11]. Alkaline hydrolysis was used to measure the total (both bound and unbound) phenolic content and an in vitro digestion simulation study was performed to predict how much of the polyphenols in the popcorn are potentially bioaccessible. The Folin–Ciocalteu assay and Ferric Reducing Ability of Plasma (FRAP) assay were used to measure total phenolics and antioxidant capacity respectively. The major difference between popcorn and other maizes is that the pericarp (skin) of the popcorn is much harder and not as porous as the pericarps of other types of maize. It is this property which allows the popcorn to pop when heated as the water inside turns to steam and the hull bursts. The skins of fruits and vegetables are known to have more polyphenols than the fruit inside [12]. Since the hull or pericarp is so much denser in popcorn than in other maizes, popcorn should contain significant amounts of polyphenols.

## 2. Materials and Methods

### 2.1. Samples

For this study, nine commercially available popcorn kernels were selected. Samples of popcorn kernels were obtained from a local grocery store or provided by popcorn companies (Brand 3 and 5). These samples were chosen to survey the total polyphenol content to see if there are differences among brands. Individual bands have been labeled 1 through 5. Different products under the same band name are denoted with a letter. Brand 3 samples are advertised as being “hulless” which means they have a softer and thinner pericarp compared to most popcorn varieties.

### 2.2. Chemicals

HPLC-Grade methanol, hexanes, anhydrous sodium acetate, Folin–Ciocalteu’s phenol reagent, 98% 2,4,6-tripyridyl-s-triazine (TPTZ), 98% (+)-catechin hydrate, butylated hydroxytoluene (BHT), bile extract (from porcine), pepsin (from porcine stomach mucosa) and pancreatin (from porcine pancreas) were purchased from Sigma Chemical Co. (St. Louis, MO, USA). Nanopure water was obtained from a Milli-Q System (Millipore Corp., Milford, MA, USA).

### 2.3. Sample Preparation

Samples were weighed and ground to a fine powder in a SPEX Sample Prep 6750 Freezer/Mill. Samples were then transferred to a glass vial and freeze dried in a Labconco FreeZone 12 Plus freeze drier, with the collector temperature set to −80 °C, overnight to remove any water present in the sample and that might have been absorbed during the grinding process. On average samples lost less than 10% of weight after the freeze-drying period. Samples were stored at −20 °C until they were further processed.

To study the difference in phenolic content and antioxidant capacity between the pericarp and the endosperm + germ, the pericarp of raw kernels were separated with a scalpel from the rest of the kernel. The pericarp and endosperm + germ were ground as described above.

To study the effect of popping, three popcorn kernel brands were popped in a household microwave oven. The popped kernels were ground into a fine powder and processed in the same manner as whole kernels.

### 2.4. Sample Treatment

#### 2.4.1. Chemical Hydrolysis

Ground popcorn samples were weighed (0.2 g) and soluble fats were extracted from the sample using hexanes. The samples were subjected to basic hydrolysis to hydrolyze any sugars or fibers covalently linked to the polyphenols using a modified protocol from Nardini [13]. 4.0 mL of methanol, 1.0 mL 0.1% ascorbic acid solution and 2.0 mL of 2.4 M NaOH was added to a sample in a plastic transfer tube and briefly vortexed. The sample was then heated for 4 h at 37 °C, with periodic mixing. It was then cooled to room temperature and 3.0 mL of 2.4 M HCl was added. The sample was then heated at 80 °C for 2 h to decompose the ascorbic acid added to the sample. Samples were stored at −20 °C until further analysis. Duplicate samples were prepared.

#### 2.4.2. In Vitro Digestion

The in vitro digestion method used was a modification of that previously described by Liu [14]. Three different brands were analyzed. 0.5 g of ground popcorn sample was mixed with 18 mL of saline (140 mM NaCl, 5 mM KCl and 150 μM BHT) and acidified to pH 2 with 0.1 M HCl. Then the sample was mixed with 0.5 mL of pepsin solution (0.2 g of pepsin in 5 mL of 0.1 M HCl) and incubated in a shaking water bath at 37 °C for 1 h. After gastric digestion, the pH of the digestate was increased to 6.9 with 0.1 M NaHCO_3_. Further intestinal-simulated digestion was performed with the addition of 2.5 mL of pancreatin-bile solution (0.240 g of bile extract and 0.040 g of pancreatin in 20.0 mL of 0.1 M NaHCO_3_) and incubated in a shaking water bath at 37 °C for 2 h. The total digestate was then acidified to pH 2 and adjusted to a total volume of 37 mL with 2.4 M HCl. Solids were filtered using gravity filtration and the digest solution was stored at −80 °C until further analysis. Duplicate samples were prepared.

### 2.5. Analysis Methods

Polyphenolic content was determined by the Folin–Ciocalteu assay while antioxidant capacity was determined by the Ferric Reducing Ability of Plasma (FRAP) assay. (+)-Catechin hydrate was used to prepare standard curves to determine the antioxidant capacity and total phenolic content of each chemically hydrolyzed sample. A Genysis 20 spectrophotometer (Thermo Scientific) was used for all absorbance readings. The Folin–Ciocalteu assay was performed by diluting Folin–Ciocalteu’s phenol reagent 5× with nanopure water. 200 µL of extract was mixed with 2000 µL of the working reagent. The assay mixture in cuvettes was allowed to react for 20 minutes at room temperature before an absorbance reading was taken at 750 nm. Total phenolic content is reported as mg catechin equivalent per gram popcorn.

The FRAP assay procedure used was a modification of the procedure described by Benzie and Strain [15]. Three reagents were prepared; a pH 3.6 acetate buffer, 10 mM TPTZ in 40 mM HCl and 20 mM FeCl_3_ in water. The working reagent was prepared by mixing the three reagents together in a 10:1:1 ratio of buffer, TPTZ and FeCl_3_. The working reagent was kept at 37 °C in a water bath. 20 µL of extract and 2000 µL of the working reagent were pipetted into cuvettes and then placed in an oven at 37 °C for 10 min before an absorbance reading was made at 593 nm. Antioxidant capacity is reported as mg catechin equivalent per gram popcorn.

### 2.6. Interference Testing

To determine possible interferences in the sample that might interact with the assays used, the chemical hydrolysis extract was run through a Polyclar VT column (GAF Chemicals, 400 μm) to remove polyphenols leaving water-soluble interfering compounds in the eluate. The procedure was adopted from Agbor [16]. The column was prepared by packing a 5 mL plastic syringe with approximately 20 mg of cotton and 300 mg of Polyclar. The column was equilibrated by running 3 mL of methanol through it followed by 2 mL of 2.4 M HCl. 3 mL of the total extraction solution was then run through the column to collect 3 mL of eluate in a 10 mL screw-capped plastic tube. The eluate was tested by the Folin–Ciocalteu and FRAP assay for non-polyphenol interferences. No interferences were found.

### 2.7. Statistical Analysis

Sigma Stat 3.0 (Jandel Scientific) was used for the statistical analysis. All samples were prepared in duplicate for each assay and the average and standard deviation were determined from 3 readings for each sample. The paired student’s *t*-test was used to determine if two data sets (i.e., popped popcorn versus raw kernel of the same sample) were significantly different from each other. The Pearson and Spearman product-moment correlation coefficient were used to analyze the agreement between the two different assays. Any result with *p* < 0.05 was deemed significant. Linear regression was used to identify trends in the data.

## 3. Results

### 3.1. Free Polyphenols

The free (unbound) antioxidant content of popcorn was analyzed by agitating 0.2 g of ground popcorn sample overnight in 10 mL of 50/50 methanol/water solution. This method gave no detectable extracted polyphenols or antioxidant capacity by either colorimetric assay. This is likely due to the polyphenols being conjugated (bound) to form an insoluble fiber. Ferulic acid, one of the major phenolic acids in corn forms cross-links with arabinoxylans and pectins [17]. Thus, in order to be absorbed by the body, the polyphenols need to be hydrolyzed in the GI tract.

### 3.2. Folin–Ciocalteu and FRAP Assays after Chemical Hydrolysis

**Figure 1:**
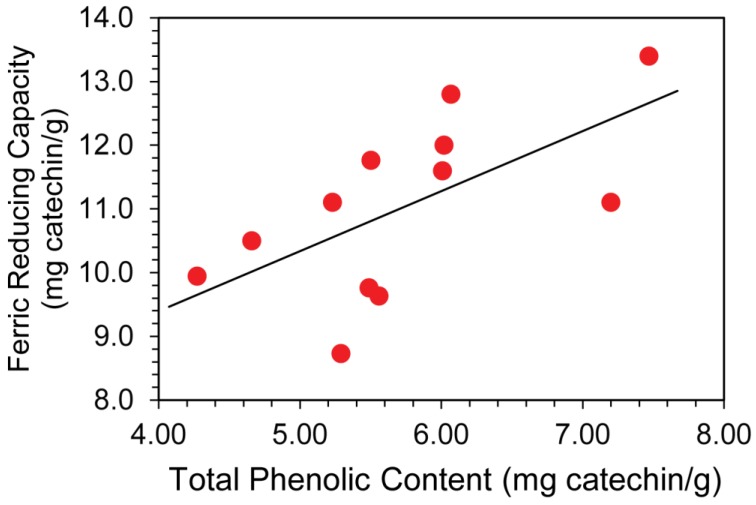
A positive correlation exists between the FRAP assay and Folin–Ciocalteu assay indicating that antioxidant capacity is closely coupled to total phenolic content, r = 0.720 (*p* < 0.05).

Total average phenolic content and antioxidant capacity for the plain, unpopped kernels is 5.93 ± 0.92 mg catechin equiv/g corn by Folin–Ciocalteu and 11.2 ± 1.5 mg catechin equiv/g corn by FRAP respectively (Table 1). The assays show a positive correlation, shown in Figure 1, for this set of data with a Pearson Correlation coefficient of r = 0.720, indicating that the total phenolic content is significantly correlated (*p* < 0.05) to the antioxidant capacity of the extract. This correlation between the FRAP and Folin–Ciocalteu assay has been observed previously in plant extracts [18]. This result indicates that the free-radical reducing capacity of popcorn is due to the presence of phenolic compounds in the kernel.

**Figure 2:**
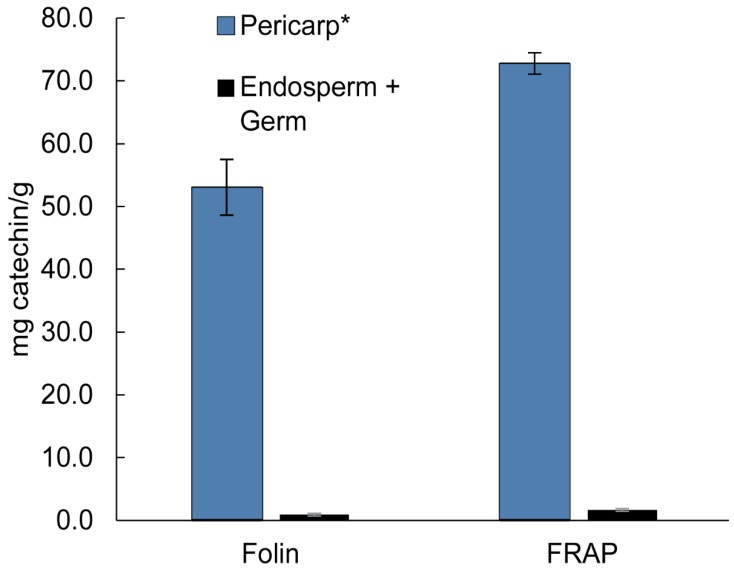
Average total phenolic content (Folin) and antioxidant capacity (FRAP) of the popcorn pericarp and endosperm + germ. * Significantly more phenolic acids are present in the pericarp versus the endosperm (n = 9, *p* < 0.05).

Additionally, the difference between the total phenolic content and antioxidant capacity of the pericarp and endosperm + germ was studied. On average, the pericarp of the raw popcorn kernel contains 98.3% of the total phenolic content and 97.8% of the antioxidant capacity of the entire kernel. Figure 2 shows the comparison between the kernel and the endosperm + germ for both the Folin–Ciocalteu and FRAP assays. The pericarp, averaged between all the brands studied, has a total phenolic content of 53.1 ± 8.8 mg catechin equiv/g pericarp and antioxidant capacity of 72.8 ± 3.4 mg catechin equiv/g pericarp. The endosperm + germ had an average total phenolic content of 0.9 ± 0.4 mg catechin equiv/g endosperm and antioxidant capacity of 1.7 ± 0.4 mg catechin equiv/g endosperm.

**Figure 3:**
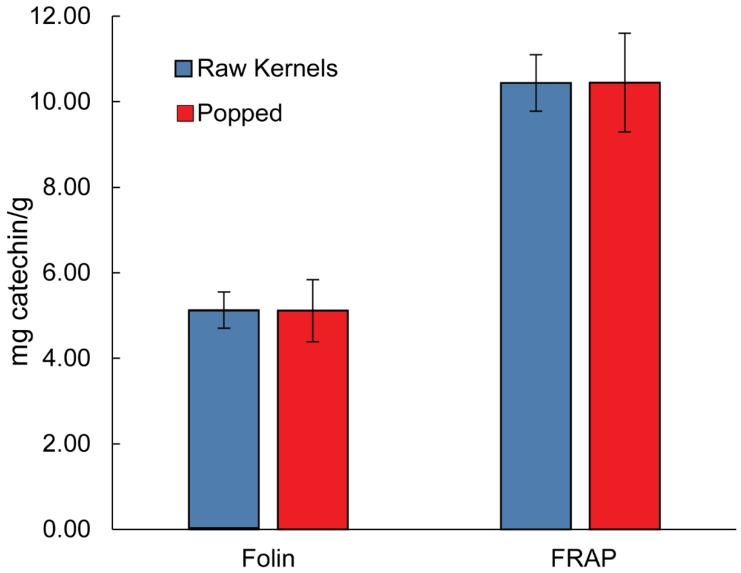
Average total phenolic content by Folin and antioxidant capacity by FRAP (mg catechin/g) of hydrolyzed raw and popped popcorn kernels. There are no significant differences between the popped and raw kernels. (n = 3, *p* > 0.05).

Since popcorn is consumed popped rather than as raw kernels, a study was performed to see whether popping had any effect on the phenolic content and antioxidant capacity. Three kernel samples from three different commercial sources were chosen for this experiment. These samples were popped using a microwave and then prepared using the sample preparation and chemical hydrolysis procedure. The Folin–Ciocalteu and FRAP assays were performed on both sets of samples. The results are summarized in Figure 3. The Student’s *t*-test for paired means was used to test for significant differences between the kernels and popped samples.

### 3.3. Folin and FRAP Assays after In Vitro Digestion

**Figure 4:**
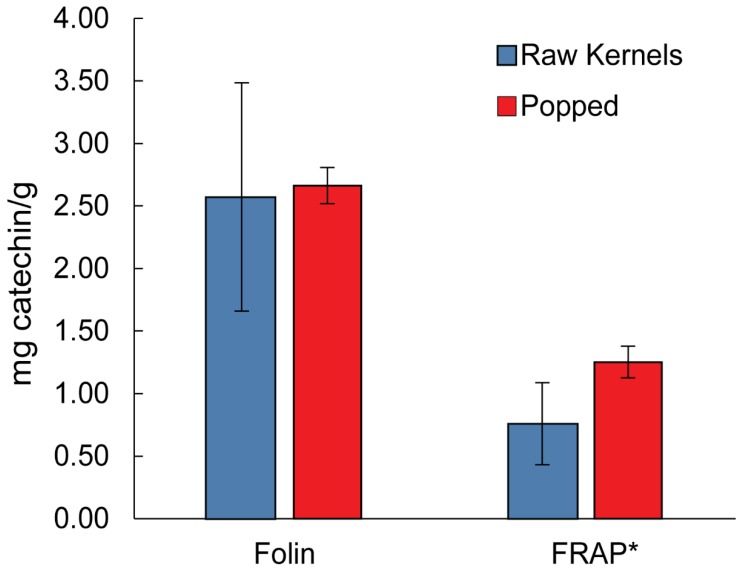
Average total phenolic content by Folin and antioxidant capacity by FRAP (mg catechin/g) of samples processed through in vitro digestion. * For antioxidant capacity, there is a significant increase in iron reducing power (n = 3, *p* < 0.05).

An in vitro digestion simulation was performed to understand how efficiently bound polyphenols in popcorn could be liberated through gastric digestion. Both raw kernels and popped kernels were analyzed to see if the popping process made any difference to the efficiency of the digestion process. The results of this study are summarized in Figure 4. Among the digested samples, the paired *t*-test shows a significant difference between popped and raw kernel samples for antioxidant capacity (*p* < 0.05) with the popped digested samples having a higher antioxidant capacity. The average antioxidant capacity is 0.76 ± 0.33 mg catechin equiv/g corn for raw kernels and 1.25 ± 0.12 mg catechin equiv/g corn for popped. For total phenolic content there does not seem to be a significant difference between popped and raw kernel samples (*p* > 0.05). The average total phenolic content is 2.57 ± 0.91 mg catechin equiv/g for raw kernels and 2.66 ± 0.15 mg catechin equiv/g for popped.

## 4. Discussion

### 4.1. Quantity of Antioxidants

Among the different grains (oats, wheat and rice), corn has been shown to contain the greatest amount of antioxidant activity due to phenolic acids [11]. However, most of the phenolic acids are bound in insoluble fiber. Dewanto et al. reported the free total phenolic content of raw sweet corn to be 0.250 ± 2.0 mg/g while a total phenolic content of 2.64 ± 0.10 mg gallic acid equiv/g was reported for corn samples extracted by basic hydrolysis (based on dry weight) [11,19]. This compares favorably with our average value of 5.93 ± 0.92 mg catechin equiv/g for basic hydrolysis and 2.66 ± 0.15 mg catechin equiv/g for in vitro digestion and supports our low value for free phenolic content. The varieties of corn used for popcorn seem to have an equal, if not slightly greater total dry phenolic content compared to other studied corn varieties. Popcorn can be consumed with little processing or preparation. According to a recent review, thermal treatments such as cooking or drying can have mixed results on the phenolic content of food depending on the cooking method and species [20]. With regard to thermal treatments, the phenolic content of grains seems to be largely unaffected by baking and the phenolic content of cooked sweet corn increases by 54% after baking [19,21]. In this work, the antioxidant capacity and total phenolic content were shown to not be significantly affected by popping in a microwave oven when the phenolics were extracted with chemical hydrolysis (*p* > 0.05) Thus, the popping process does not appear to degrade the phenolic compounds originally present in the kernel. However, when extracted through in vitro digestion, the popped kernels had significantly more antioxidant capacity compared to raw kernels.

In vitro digestion has been shown to increase total phenolic content in cooked foods. For example, recently Ti et al. showed an increase in total phenolic content and antioxidant capacity of cooked brown rice after in vitro digestion[22]. Generally it is thought that cooking or thermal processing breaks down the cell wall, thus freeing any bound phenolic compounds [19,22]. This would lead to increased extraction, especially for enzymatic digestion as we observed.

### 4.2. Popcorn as a Source of Dietary Antioxidants

Since popcorn is a popular whole grain snack food, it may be a significant potential source of phytochemicals in the American diet [6]. In fact, of the total amount of whole grains Americans consume, 17% comes from popcorn [23]. Popcorn consumers also have 250% higher whole grain intake and 22% higher intake of fiber versus non-popcorn consumers [6]. The per capita intake of total polyphenols in the average US diet is about 2000 mg by our data for foods and beverages in the US (Vinson, unpublished results). If we consider that the average popcorn consumer consumes 39 g of popcorn per day [6], then this consumer is taking in an average of approximately 240 mg (12%) of their total dietary polyphenols from popcorn based upon our values for total polyphenols as measured by the Folin–Ciocalteu assay.

In this study popcorn has been found to contain a substantial quantity of polyphenols. Furthermore, our in vitro digestion study indicates up to 50% of the total polyphenols in popcorn that is ingested has the potential to be bioavailable. Fiber bound ferulic acid is already known to be bioavailable in mammals via esterases with activity towards ester bound hydroxycinnamates [24]. In addition, it has been shown in humans that ferulic acid is liberated from whole grain during the digestion process and makes its way into the body [25]. Consumption of ferulic acid in animals has been shown to act to lower oxidative stress, increase insulin, decrease lipids and atherosclerosis and reduce blood pressure [26]. Thus, the reduction of risk of diabetes, heart disease and hypertension may accrue from consuming ferulate in whole grains such as popcorn. However, it is crucial that the entire kernel be consumed. Approximately 98% of the phenolic content and antioxidant content is in the pericarp, despite it being only 15–20% of the total weight of the popcorn kernel. Sweet corn has a similar weight distribution, with 20% of the kernel weight being the pericarp [11]. This agrees with previous studies on maizes and their antioxidant capacity [19,27]. Corn bran is among the highest antioxidant food in existence.

The popping process does not have any substantial effect on the phenolic content in popcorn, making it an ideal source of unprocessed grains and polyphenols. In fact, popcorn eaten plain and air popped is the only food that is 100% whole grain by weight. However it is often consumed with added fat and salt, ingredients which when eaten excessively could negatively affect diet and health [28,29]. The popularity and availability of popcorn makes it a possible source of a large portion of the daily antioxidant intake along with other sources such as fruits and beverages. Popcorn is also the most satiating food among the snacks and desserts [30]. For example, it is 1.6 times more satiating than potato chips, a snack high in acrylamide which is shown to increase oxidative stress and inflammation in humans [31,32]. Thus, whole grain popcorn is a prudent choice for those wanting to reduce feelings of hunger while managing energy intake and ultimately, body weight. For reference, a cup of air popped popcorn only contains about 30 Calories. Furthermore coupled with seasonings such as spices and nuts, popcorn can become an even more ideal snack since nuts and spices have large quantities of antioxidants themselves [33]. Therefore, of the available snack foods, popcorn certainly has the potential to provide the most health benefits as part of a balanced diet.

## Figures and Tables

**Table 1:** Total phenolic content and antioxidant capacity of raw kernel samples found by chemical basic hydrolysis. Quantities are reported as mg catechin equivalent per gram. Results are mean ± standard deviation, n = 2.

Sample	Folin Assay (mg catechin/g)	FRAP Assay (mg catechin/g)
brand 1	4.66 ± 0.31	10.5 ± 0.4
brand 2	5.23 ± 0.27	11.1 ± 0.5
brand 3A	5.49 ± 0.10	9.76 ± 0.15
brand 3B	6.01 ± 0.04	11.6 ± 0.2
brand 3C	5.29 ± 0.19	8.73 ± 0.12
brand 3D	6.02 ± 0.10	12.0 ± 0.2
brand 4	6.07 ± 0.02	12.8 ± 0.5
brand 5A	7.20 ± 0.04	11.1 ± 0.9
brand 5B	7.47 ± 0.05	13.4 ± 0.1
Average	5.93 ± 0.92	11.2 ± 1.5
